# Comparison of eye movements in schizophrenia and autism spectrum disorder

**DOI:** 10.1002/npr2.12085

**Published:** 2019-11-27

**Authors:** Tomoko Shiino, Kenichiro Miura, Michiko Fujimoto, Noriko Kudo, Hidenaga Yamamori, Yuka Yasuda, Manabu Ikeda, Ryota Hashimoto

**Affiliations:** ^1^ Department of Pathology of Mental Diseases National Institute of Mental Health National Center of Neurology and Psychiatry Kodaira Japan; ^2^ United Graduate School of Child Development Osaka University Suita Japan; ^3^ Division of Psychosocial Support for Nurturing Research Center for Child Mental Development University of Fukui Eiheiji Japan; ^4^ Department of Integrative Brain Science Graduate School of Medicine Kyoto University Kyoto Japan; ^5^ Department of Psychiatry Graduate School of Medicine Osaka University Suita Japan; ^6^ Japan Community Health care Organization Osaka Hospital Osaka Japan; ^7^ Life Grow Brilliant Mental Clinic Osaka Japan

**Keywords:** autism spectrum disorder, eye movement, free viewing, schizophrenia, smooth pursuit

## Abstract

**Aim:**

Eye movement abnormalities are often associated with psychiatric illness. Subjects with either schizophrenia or autism spectrum disorder (ASD) have been reported to show eye movement abnormalities. However, it is still unclear whether eye movement abnormalities in schizophrenia and in ASD have common features. This study aimed to understand the similarities/differences in eye movement abnormalities of subjects with schizophrenia and those with ASD.

**Methods:**

We analyzed 75 eye movement characteristics of 83 subjects with schizophrenia, 17 subjects with ASD and 255 healthy controls that were collected during fixation, smooth pursuit and free viewing tasks using analysis of covariance with the covariates age and sex.

**Results:**

We found significant effects across groups on 21 eye movement characteristics, of which 4 characteristics had large effect sizes. Post hoc multiple comparisons indicated significant differences between the subjects with schizophrenia and healthy controls across all 21 characteristics. On the other hand, no significant difference between the ASD group and healthy control group was found. Instead, the subjects with ASD showed significant differences from the subjects with schizophrenia in 5 eye movement characteristics during the free viewing and smooth pursuit eye movements.

**Conclusions:**

The present results suggest that eye movement abnormalities in the subjects with ASD are different from those with schizophrenia and that the tasks in this study are suitable to detect eye movement abnormality in schizophrenia. Thus, the eye movement examinations used here may distinguish subjects with schizophrenia from those with ASD.

## Abstract 2

This study aimed to understand the similarities/differences in eye movement abnormalities of subjects with schizophrenia and those with ASD. The subjects with ASD showed significant differences from the subjects with schizophrenia in five characteristics during the free viewing and smooth pursuit eye movements. The results suggest that eye movement abnormalities in the subjects with ASD are different from those with schizophrenia.

## Graphical abstract

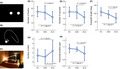

## 1 INTRODUCTION

Discovery of disease‐specific biomarkers or behavioral indicators with sufficient sensitivity is an important issue to improve the clinical diagnosis of mental illness. Magnetic resonance images, near‐infrared spectroscopy, genetic markers, cognitive functions, and eye movements are candidates.^1^, ^2^, ^3^, ^4^, ^5^, ^6^ Of them, eye movement is representative of sensorimotor functions of the brain, and eye movement abnormalities have been reported in subjects with schizophrenia.^7^, ^8^, ^9^ It has been frequently reported that subjects with schizophrenia can be successfully distinguished from healthy individuals by using eye movements.^4^, ^10^, ^11^, ^12^, ^13^ However, it is unknown whether the eye movement abnormalities that distinguish subjects with schizophrenia from healthy individuals are also effective in discrimination from other psychiatric disorders, such as autism spectrum disorder (ASD), which is a neurodevelopmental condition in which affected individuals have difficulties in social interactions and communications.^14^ Eye movement abnormalities in ASD have also been widely described.^15^, ^16^, ^17^, ^18^, ^19^ In addition, it has been shown that subjects with ASD can be distinguished from typically developing subjects by using eye movements.^20^ The purpose of the present study is to understand the similarities/differences in eye movement abnormalities between subjects with schizophrenia and those with ASD. Once different abnormalities between schizophrenia and ASD are established, such abnormalities would be useful for the differential diagnosis between these psychiatric disorders. To date, eye movement abnormalities in different mental disorders have been examined using different methodologies to emphasize the characteristics of a particular disorder. In the present study, we compared the eye movement characteristics of patients with schizophrenia and ASD that were collected by using exactly the same procedures.

## 2 METHODS

The details of the subject inclusion, eye movement examination, and data collection have been described elsewhere,^4^ so we will describe them only briefly.

We studied the eye movement characteristics of 83 subjects with schizophrenia, 17 subjects with ASD, and 255 healthy controls with no history of psychiatric illness, whose demographic information is summarized in Table S1. The patients were recruited at the Osaka University Hospital and had been diagnosed according to the criteria from the Diagnostic and Statistical Manual of Mental Disorders, Fourth Edition (DSM‐IV) based on the Structured Clinical Interview for DSM‐IV (SCID). The study was performed in accordance with the World Medical Association's Declaration of Helsinki and was approved by the Research Ethical Committee of Osaka University. All participants provided written consent to the study after a full explanation of the study procedures. Anonymity was preserved for all participants.

**Figure 1:**
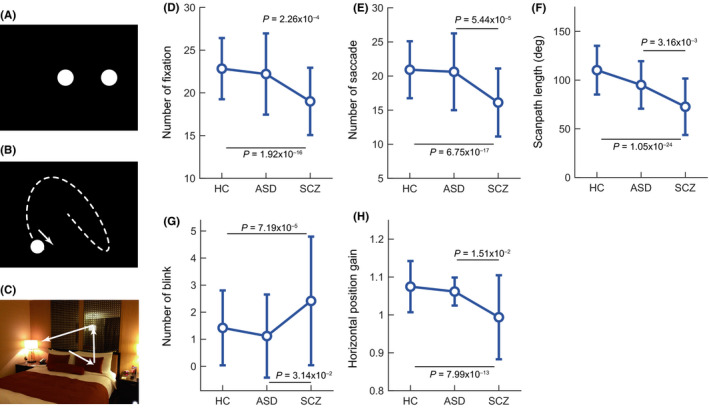
Schematic diagrams of stimulus paradigms ((A): fixation task, (B): smooth pursuit task [Lissajous], (C): free viewing task) and comparisons among groups in five characteristics (number of fixations (D), number of saccades (E), scanpath length (F), and number of blinks (G) in the free viewing eye movements and the horizontal position gain (H) in the smooth pursuit eye movements [fast Lissajous]). Error bars indicate standard deviations. The Bonferroni‐adjusted *P*‐values obtained from the post hoc multiple comparisons are described when the difference is statistically significant

Seventy‐five eye movement characteristics extracted from the eye movement data recorded during conditions in which the subjects performed fixation (Figure 1A), smooth pursuit (Figure 1B), and free viewing tasks (Figure 1C), which have been described elsewhere,^4^ were analyzed. The statistical analyses were performed using Statistical Package for the Social Sciences (SPSS) version 25 (IBM Corp., Armonk, NY). Group comparisons of the eye movement characteristics were performed using analysis of covariance (ANCOVA) with the covariates age and sex. Multiple comparisons with Bonferroni corrections were carried out to examine differences between diagnostic groups.

## 3 RESULTS

In the group comparisons, we found significant effects on 21 eye movement characteristics after Bonferroni corrections (*P* < .05/75, Table S2), of which four eye movement characteristics involving the number of fixations, number of saccades, scanpath length, and fixation density in the free viewing eye movements had large effect sizes (partial η^2^ > 0.16). The multiple comparisons with Bonferroni corrections on all 21 eye movement characteristics indicated significant differences between subjects with schizophrenia and healthy controls (Table S3). On the other hand, no significant difference was found in any of the 21 eye movement characteristics in comparisons between the subjects with ASD and healthy controls. Instead, the subjects with ASD showed significant differences from those with schizophrenia in the 5 of 21 eye movement characteristics involving the number of fixations, number of saccades, scanpath length, and number of blinks in the free viewing eye movements and the horizontal position gain in the smooth pursuit eye movements (fast Lissajous) (Figure 1 and Table S3).

## 4 DISCUSSION

In this study, we compared the eye movement characteristics of the subjects with schizophrenia and ASD that were collected using identical tests and data analyses. The eye movement characteristics that successfully represented eye movement disorders of the subjects with schizophrenia did not show abnormalities in the subjects with ASD. This might not be simply due to the small sample size of the subjects with ASD, but due to smaller differences between the subjects with ASD and healthy control, because the values of the eye movement characteristics in the ASD group were generally similar to those in the healthy controls or intermediate between the healthy controls and those with schizophrenia (see Table S2). These results suggest that eye movement abnormalities of the subjects with ASD were not severe as schizophrenia, at least in terms of the characteristics examined here.

Gaze behaviors in ASD were known to be different from typically developing subjects when they watched visual stimuli such as social scenes from a film.^17^, classroom video.^16^, and facial stimuli.^21^. In contrast, we found no abnormalities in the subjects with ASD. This discrepancy might have been due to a difference in the observed visual stimuli during the free viewing eye movements, most of the visual stimuli used in this study were not associated with social situations.^4^ Thus, the visual stimuli representing social situations and appropriate measures to characterize the gaze behaviors might be required to induce abnormalities in ASD. On the other hand, we found clear abnormalities in the gaze behaviors of the subjects with schizophrenia, which was consistent with previous studies.^4^, ^22^ The number of saccades and scanpath length were smaller than the ASD subjects, suggesting lower scanning activities in subjects with schizophrenia than in those with ASD. The examination of gaze behaviors using nonsocial visual stimuli may help in the differential diagnosis between schizophrenia and ASD. In addition, we found no abnormality in the smooth pursuit performance in the subjects with ASD, whereas the subjects with schizophrenia showed robust performance deficits in the smooth pursuit examinations used here. The horizontal position gain was smaller in the subjects with schizophrenia than in the subjects with ASD, suggesting lower tracking performance in schizophrenia. This characteristic may also help distinguish the subjects with schizophrenia from those with ASD.

In summary, our present results suggest that these eye movement characteristics would help objectively distinguish the subjects with schizophrenia from those with ASD who are sometimes difficult to discriminate between with symptom‐based criteria from the onset. Future replication studies with larger sample sizes should be carried out to establish these eye movement characteristics as biomarkers to distinguish subjects with schizophrenia from those with ASD.

## CONFLICT OF INTEREST

The authors declare no conflict of interest.

## AUTHOR CONTRIBUTIONS

TS was critically involved in the analysis of the data and wrote the first draft of the manuscript. KM was critically involved in the study design and analysis of the data and contributed to the interpretation of the data and the writing of the manuscript. MF, NK, HY, and YY were involved in the subject recruitment process, the clinical diagnostic assessments, and contributed to the data interpretation. MI contributed to the interpretation of the data and the writing of the manuscript. RH supervised the entire project, collected the data, and was critically involved in the design and interpretation of the data. All authors contributed to and approved the final manuscript.

## ETHICAL APPROVAL

The study was performed in accordance with the World Medical Association's Declaration of Helsinki and was approved by the Research Ethical Committee of Osaka University. Anonymity was preserved for all participants.

## INFORMED CONSENT

All participants provided written consent to the study after a full explanation of the study procedures.

## Data Availability

The data sets generated during and/or analyzed during the current study are not publicly available because they contain information that could compromise research participant privacy/consent.
