# Supplementary material for: Comparison of eye movements in schizophrenia and autism spectrum disorder
Source: Neuropsychopharmacol Rep. 2019 Nov 27;40(1):92–5. doi: 10.1002/npr2.12085 (PMC7292215; doi:10.1002/npr2.12085)
Supplement: Supplementary file 1 [file NPR2-40-92-s001.docx]

| Table S1. Demographical and clinical characteristics of subjects. | | | | |  |  |  |  |  |  |  |  |  |  |
| --- | --- | --- | --- | --- | --- | --- | --- | --- | --- | --- | --- | --- | --- | --- |
|  | HC (n=255) | | |  | SCZ (n=83) | | |  | ASD (n=17) | | |  | statistic | p-value |
| Age | 28.74 | ± | 11.47 |  | 35.39 | ± | 12.65 |  | 22.00 | ± | 4.78 |  | 14.65 | 7.76×10^-7^ |
| Gender | 145/110 | | |  | 42/41 | | |  | 9/8 | | |  | 1.03 | 5.98×10^-1^ |
| Education years | 14.98 | ± | 1.80 |  | 13.76 | ± | 2.60 |  | 12.18 | ± | 2.10 |  | 23.80 | 2.02×10^-10^ |
| WAIS-III FIQ | 116.48 | ± | 10.27 | (252) | 88.83 | ± | 17.35 | (76) | 102.53 | ± | 16.93 |  | 145.25 | 2.17×10^-46^ |
| JART-25 | 110.11 | ± | 6.17 | (250) | 101.16 | ± | 9.85 | (76) | 107.96 | ± | 8.61 | (14) | 44.35 | 8.00×10^-18^ |
| Onset age (years) |  |  |  |  | 24.29 | ± | 11.38 |  | 12.36 | ± | 6.66 | (14) | 3.80 | 2.53×10^-4^ |
| Duration of illness (years) |  |  |  |  | 11.07 | ± | 9.45 |  | 9.93 | ± | 5.89 | (14) | 0.44 | 6.63×10^-1^ |
| PANSS_total |  |  |  |  | 83.80 | ± | 23.34 | (81) |  |  |  |  |  |  |
| Daily antipsychotic dose (mg/day) |  |  |  |  | 604.60 | ± | 543.35 |  |  |  |  |  |  |  |
| Autism Spectrum Quotient |  |  |  |  |  |  |  |  | 31.53 | ± | 8.19 |  |  |  |

Mean ± standard deviation (SD) and the results of statistical tests are shown. Daily antipsychotic dosages were converted to chlorpromazine equivalents (mg/day). Group difference in gender was tested using the χ2 test, onset age and duration of illness using t-test, and ANOVA was used elsewhere. The figures in parentheses are the numbers of samples available (described if there are missing values). Abbreviations WAIS-III: Wechsler adult intelligence scale - third edition (Japanese version) ; FIQ: Full scale intelligence quotient; JART: Japanese version of the national adult reading test; PANSS: Positive and negative syndrome scale; HC: Healthy controls; SCZ: Subjects with schizophrenia; ASD: Subjects with autism spectrum disorder.

| Table S2: Results of ANCOVA | |  |  |  |  |  |  |  |  |  |  |  |  |
| --- | --- | --- | --- | --- | --- | --- | --- | --- | --- | --- | --- | --- | --- |
|  |  | HC (n=255) | | | SCZ (n=83) | | | ASD (n=17) | | | ANCOVA statistics | | |
|  |  |  |  |  |  |  |  |  |  |  | f-value | p-value | partial η2 |
| **Free viewing test** | |  |  |  |  |  |  |  |  |  |  |  |  |
|  | **Number of fixations** | **22.83** | **±** | **3.58** | **19.01** | **±** | **3.94** | **22.21** | **±** | **4.75** | **38.90** | **5.57×10^-16^** | **0.182** |
|  | **Duration of fixation** | **249.24** | **±** | **40.16** | **292.31** | **±** | **67.67** | **269.53** | **±** | **62.10** | **26.14** | **2.62×10^-11^** | **0.130** |
|  | **Number of saccades** | **20.92** | **±** | **4.18** | **16.11** | **±** | **4.99** | **20.62** | **±** | **5.63** | **40.40** | **1.64×10^-16^** | **0.188** |
|  | Duration of saccades | 39.75 | ± | 5.01 | 37.69 | ± | 6.55 | 38.04 | ± | 4.66 | 4.94 | 7.66×10^-3^ | 0.027 |
|  | Saccade amplitude | 3.50 | ± | 1.12 | 2.92 | ± | 1.19 | 3.08 | ± | 1.11 | 7.42 | 6.96×10^-4^ | 0.041 |
|  | Average saccade velocity | 85.93 | ± | 19.97 | 75.51 | ± | 22.13 | 81.59 | ± | 20.39 | 6.78 | 1.29×10^-3^ | 0.037 |
|  | Peak saccade velocity | 160.65 | ± | 44.88 | 155.69 | ± | 49.82 | 150.60 | ± | 37.85 | 1.09 | 3.39×10^-1^ | 0.006 |
|  | **Scanpath length** | **110.20** | **±** | **25.03** | **72.66** | **±** | **28.96** | **95.05** | **±** | **24.39** | **63.71** | **2.53×10^-24^** | **0.267** |
|  | **Fixation density** | **1.11** | **±** | **0.29** | **1.56** | **±** | **0.63** | **1.30** | **±** | **0.38** | **36.01** | **6.01×10^-15^** | **0.171** |
|  | Main sequence *a* | 467.66 | ± | 117.80 | 479.51 | ± | 116.68 | 511.22 | ± | 106.45 | 1.63 | 1.97×10^-1^ | 0.009 |
|  | Main sequence *b* | 10.64 | ± | 3.93 | 9.75 | ± | 4.83 | 12.36 | ± | 4.42 | 0.57 | 5.67×10^-1^ | 0.003 |
|  | Main sequence *c* | 29.93 | ± | 7.38 | 28.76 | ± | 9.75 | 33.29 | ± | 5.61 | 0.93 | 3.94×10^-1^ | 0.005 |
|  | **Number of blinks** | **1.42** | **±** | **1.38** | **2.42** | **±** | **2.38** | **1.12** | **±** | **1.54** | **9.64** | **8.39×10^-5^** | **0.052** |
|  |  |  |  |  |  |  |  |  |  |  |  |  |  |
| **Smooth pursuit test** | |  |  |  |  |  |  |  |  |  |  |  |  |
| ***Horizontal pursuit*** | |  |  |  |  |  |  |  |  |  |  |  |  |
|  | SNR | 1.81 | ± | 0.25 | 1.85 | ± | 0.26 | 1.76 | ± | 0.30 | 2.31 | 1.01×10^-1^ | 0.013 |
|  | **Horizontal position gain** | **1.06** | **±** | **0.07** | **1.02** | **±** | **0.09** | **1.05** | **±** | **0.07** | **10.58** | **3.47×10^-5^** | **0.057** |
|  | Horizontal RMSE | 20.29 | ± | 15.43 | 19.33 | ± | 17.06 | 20.02 | ± | 13.39 | 0.51 | 5.98×10^-1^ | 0.003 |
|  | Number of fixations | 56.52 | ± | 17.06 | 50.16 | ± | 15.14 | 55.74 | ± | 15.76 | 3.78 | 2.39×10^-2^ | 0.021 |
|  | Duration of fixation | 267.68 | ± | 106.28 | 299.22 | ± | 114.40 | 285.84 | ± | 146.49 | 2.36 | 9.62×10^-2^ | 0.013 |
|  | Number of saccades | 55.94 | ± | 19.93 | 47.52 | ± | 17.24 | 54.88 | ± | 17.35 | 4.67 | 9.96×10^-3^ | 0.026 |
|  | Duration of saccades | 27.33 | ± | 5.68 | 29.22 | ± | 6.59 | 27.03 | ± | 5.21 | 0.50 | 6.10×10^-1^ | 0.003 |
|  | Saccade amplitude | 1.73 | ± | 0.70 | 1.76 | ± | 0.73 | 1.81 | ± | 0.73 | 0.52 | 5.96×10^-1^ | 0.003 |
|  | Average saccade velocity | 60.76 | ± | 14.97 | 60.27 | ± | 17.44 | 63.58 | ± | 16.34 | 0.40 | 6.72×10^-1^ | 0.002 |
|  | Peak saccade velocity | 88.48 | ± | 41.33 | 109.43 | ± | 48.67 | 94.68 | ± | 37.18 | 3.27 | 3.90×10^-2^ | 0.018 |
|  | Horizontal velocity gain | 0.85 | ± | 0.13 | 0.82 | ± | 0.16 | 0.86 | ± | 0.10 | 0.58 | 5.61×10^-1^ | 0.003 |
|  | **Number of blinks** | **2.58** | **±** | **2.51** | **4.39** | **±** | **5.56** | **2.06** | **±** | **2.50** | **8.09** | **3.69×10^-4^** | **0.044** |
| ***Slow Lissajous*** | |  |  |  |  |  |  |  |  |  |  |  |  |
|  | Horizontal SNR | 2.11 | ± | 0.14 | 2.15 | ± | 0.18 | 2.10 | ± | 0.13 | 2.53 | 8.09×10^-2^ | 0.014 |
|  | **Horizontal position gain** | **1.07** | **±** | **0.05** | **1.03** | **±** | **0.05** | **1.05** | **±** | **0.04** | **15.40** | **3.87×10^-7^** | **0.081** |
|  | Horizontal RMSE | 11.84 | ± | 7.04 | 10.21 | ± | 6.65 | 11.72 | ± | 4.68 | 1.12 | 3.28×10^-1^ | 0.006 |
|  | Vertical SNR | 1.98 | ± | 0.22 | 2.03 | ± | 0.22 | 1.93 | ± | 0.20 | 1.31 | 2.72×10^-1^ | 0.007 |
|  | Vertical position gain | 1.05 | ± | 0.13 | 1.01 | ± | 0.09 | 1.03 | ± | 0.10 | 1.86 | 1.57×10^-1^ | 0.011 |
|  | Vertical RMSE | 21.87 | ± | 14.42 | 19.04 | ± | 13.62 | 22.52 | ± | 8.91 | 0.63 | 5.33×10^-1^ | 0.004 |
|  | Number of fixations | 48.11 | ± | 16.27 | 41.15 | ± | 13.15 | 47.26 | ± | 10.80 | 7.25 | 8.23×10^-4^ | 0.040 |
|  | Duration of fixations | 310.05 | ± | 138.59 | 360.33 | ± | 140.20 | 304.00 | ± | 101.37 | 5.10 | 6.58×10^-3^ | 0.028 |
|  | **Number of saccades** | **46.46** | **±** | **18.66** | **37.51** | **±** | **14.89** | **45.85** | **±** | **12.75** | **8.46** | **2.59×10^-4^** | **0.046** |
|  | Duration of saccades | 24.28 | ± | 4.56 | 26.19 | ± | 5.13 | 24.78 | ± | 4.02 | 2.25 | 1.07×10^-1^ | 0.013 |
|  | Saccade amplitude | 1.24 | ± | 0.38 | 1.31 | ± | 0.44 | 1.33 | ± | 0.56 | 0.87 | 4.21×10^-1^ | 0.005 |
|  | Average saccade velocity | 50.17 | ± | 8.25 | 50.01 | ± | 11.13 | 52.14 | ± | 12.09 | 0.40 | 6.68×10^-1^ | 0.002 |
|  | **Peak saccade velocity** | **64.41** | **±** | **24.03** | **80.62** | **±** | **29.91** | **71.97** | **±** | **24.92** | **7.64** | **5.64×10^-4^** | **0.042** |
|  | **Horizontal velocity gain** | **0.95** | **±** | **0.10** | **0.89** | **±** | **0.13** | **0.93** | **±** | **0.08** | **9.41** | **1.04×10^-4^** | **0.051** |
|  | Vertical velocity gain | 0.92 | ± | 0.13 | 0.89 | ± | 0.19 | 0.88 | ± | 0.13 | 0.87 | 4.21×10^-1^ | 0.005 |
|  | Number of blinks | 3.16 | ± | 2.92 | 4.67 | ± | 5.40 | 2.71 | ± | 2.57 | 5.18 | 6.06×10^-3^ | 0.029 |
| ***Fast Lissajous*** | |  |  |  |  |  |  |  |  |  |  |  |  |
|  | Horizontal SNR | 1.93 | ± | 0.14 | 1.95 | ± | 0.17 | 1.94 | ± | 0.15 | 0.52 | 5.97×10^-1^ | 0.003 |
|  | **Horizontal position gain** | **1.07** | **±** | **0.07** | **0.99** | **±** | **0.11** | **1.06** | **±** | **0.04** | **28.93** | **2.36×10^-12^** | **0.142** |
|  | Horizontal RMSE | 14.78 | ± | 8.77 | 13.42 | ± | 9.70 | 12.83 | ± | 4.60 | 0.87 | 4.19×10^-1^ | 0.005 |
|  | Vertical SNR | 1.73 | ± | 0.19 | 1.77 | ± | 0.21 | 1.74 | ± | 0.22 | 0.97 | 3.78×10^-1^ | 0.006 |
|  | **Vertical position gain** | **1.03** | **±** | **0.15** | **0.93** | **±** | **0.15** | **1.02** | **±** | **0.13** | **10.91** | **2.54×10^-5^** | **0.059** |
|  | Vertical RMSE | 25.80 | ± | 15.84 | 23.60 | ± | 15.37 | 26.21 | ± | 14.03 | 0.29 | 7.45×10^-1^ | 0.002 |
|  | **Number of fixations** | **72.84** | **±** | **16.18** | **61.73** | **±** | **14.34** | **65.53** | **±** | **15.06** | **14.65** | **7.79×10^-7^** | **0.077** |
|  | **Duration of fixations** | **197.13** | **±** | **65.83** | **238.80** | **±** | **80.45** | **238.19** | **±** | **82.51** | **11.56** | **1.37×10^-5^** | **0.062** |
|  | **Number of saccades** | **74.86** | **±** | **20.42** | **60.43** | **±** | **18.35** | **67.59** | **±** | **17.97** | **14.40** | **9.74×10^-7^** | **0.076** |
|  | Duration of saccades | 31.37 | ± | 5.21 | 32.02 | ± | 5.60 | 30.51 | ± | 5.70 | 0.05 | 9.56×10^-1^ | 2.6×10^-4^ |
|  | Saccade amplitude | 2.02 | ± | 0.61 | 2.10 | ± | 0.71 | 2.00 | ± | 0.74 | 0.06 | 9.45×10^-1^ | 3.2×10^-4^ |
|  | Average saccade velocity | 60.88 | ± | 12.93 | 62.29 | ± | 14.11 | 60.71 | ± | 12.80 | 0.06 | 9.45×10^-1^ | 3.3×10^-4^ |
|  | Peak saccade velocity | 81.84 | ± | 36.59 | 102.11 | ± | 45.39 | 87.35 | ± | 37.48 | 4.33 | 1.38×10^-2^ | 0.024 |
|  | Horizontal velocity gain | 0.84 | ± | 0.13 | 0.77 | ± | 0.17 | 0.85 | ± | 0.08 | 6.72 | 1.37×10^-3^ | 0.037 |
|  | Vertical velocity gain | 0.77 | ± | 0.15 | 0.70 | ± | 0.18 | 0.79 | ± | 0.15 | 4.51 | 1.16×10^-2^ | 0.025 |
|  | Number of blinks | 3.15 | ± | 3.23 | 4.62 | ± | 5.55 | 2.62 | ± | 2.41 | 4.10 | 1.74×10^-2^ | 0.023 |
|  |  |  |  |  |  |  |  |  |  |  |  |  |  |
| **Fixation test** | |  |  |  |  |  |  |  |  |  |  |  |  |
| ***No distractor*** | |  |  |  |  |  |  |  |  |  |  |  |  |
|  | Number of fixations | 4.13 | ± | 4.24 | 5.52 | ± | 4.97 | 4.15 | ± | 4.37 | 2.56 | 7.91×10^-2^ | 0.014 |
|  | Duration of fixation | 2391.7 | ± | 1675.8 | 1755.6 | ± | 1464.0 | 2327.7 | ± | 1675.9 | 2.46 | 8.73×10^-2^ | 0.014 |
|  | Number of fixations | 2.94 | ± | 4.54 | 3.92 | ± | 5.27 | 2.91 | ± | 4.56 | 1.08 | 3.42×10^-1^ | 0.006 |
|  | Scanpath length | 2.11 | ± | 4.22 | 3.42 | ± | 5.07 | 2.26 | ± | 3.75 | 2.20 | 1.12×10^-1^ | 0.012 |
|  | Number of microsaccades | 7.07 | ± | 4.83 | 6.49 | ± | 4.88 | 4.56 | ± | 2.97 | 2.62 | 7.43×10^-2^ | 0.015 |
|  | **Number of blinks** | **0.39** | **±** | **0.66** | **0.92** | **±** | **1.42** | **0.50** | **±** | **0.90** | **9.49** | **9.73×10^-5^** | **0.051** |
| ***Near distractor*** | |  |  |  |  |  |  |  |  |  |  |  |  |
|  | Number of fixations | 4.25 | ± | 3.77 | 5.29 | ± | 4.00 | 5.35 | ± | 3.85 | 2.41 | 9.16×10^-2^ | 0.014 |
|  | Duration of fixation | 2235.0 | ± | 1539.2 | 1514.3 | ± | 1297.9 | 1525.1 | ± | 1152.6 | 6.61 | 1.52×10^-3^ | 0.036 |
|  | Number of fixations | 3.09 | ± | 4.01 | 3.81 | ± | 4.30 | 4.26 | ± | 4.35 | 1.34 | 2.63×10^-1^ | 0.008 |
|  | Scanpath length | 2.32 | ± | 3.00 | 3.61 | ± | 5.04 | 3.40 | ± | 4.46 | 3.63 | 2.76×10^-2^ | 0.020 |
|  | Number of microsaccades | 6.55 | ± | 4.29 | 6.27 | ± | 4.73 | 4.15 | ± | 3.85 | 2.39 | 9.33×10^-2^ | 0.013 |
|  | **Number of blinks** | **0.35** | **±** | **0.69** | **0.82** | **±** | **1.27** | **0.38** | **±** | **0.47** | **9.01** | **1.53×10^-4^** | **0.049** |
| ***Far distractor*** | |  |  |  |  |  |  |  |  |  |  |  |  |
|  | Number of fixations | 4.31 | ± | 4.02 | 5.79 | ± | 4.92 | 4.75 | ± | 3.09 | 3.04 | 4.92×10^-2^ | 0.017 |
|  | **Duration of fixation** | **2271.7** | **±** | **1547.0** | **1424.6** | **±** | **1205.6** | **1695.6** | **±** | **1295.5** | **8.60** | **2.26×10^-4^** | **0.047** |
|  | Number of saccades | 3.16 | ± | 4.14 | 4.30 | ± | 5.35 | 3.44 | ± | 3.08 | 1.65 | 1.93×10^-1^ | 0.009 |
|  | Scanpath length | 2.51 | ± | 3.39 | 4.25 | ± | 5.61 | 4.16 | ± | 4.83 | 5.52 | 4.37×10^-3^ | 0.031 |
|  | Number of microsaccades | 6.95 | ± | 4.64 | 6.23 | ± | 4.45 | 4.25 | ± | 2.98 | 3.46 | 3.25×10^-2^ | 0.019 |
|  | **Number of blinks** | **0.35** | **±** | **0.64** | **0.94** | **±** | **1.38** | **0.50** | **±** | **0.50** | **12.66** | **4.90×10^-6^** | **0.067** |

Mean ± SD and ANCOVA statistics are shown. The names of eye movement tests are written in bold and underlined script, paradigms within the tests are written in bold and italic script, and the individual measures are written in smaller plain text. Raw *p* values are shown. The measures with significant effects of subject’s group after Bonferroni correction (*p* < 0.05/75) are shown in Bold script. HC: Healthy controls; SCZ: Subjects with schizophrenia; ASD: Subjects with autism spectrum disorder; SNR: Signal-to-noise ratio; RMSE: Root mean square error.

| Table S3: Results of multiple comparison (p-value) | | | | | | |
| --- | --- | --- | --- | --- | --- | --- |
|  |  | SCZ vs HC |  | ASD vs HC |  | SCZ vs ASD |
| **Free viewing test** | |  |  |  |  |  |
|  | **Number of fixations** | **1.92×10^-16^** |  | 1.00 |  | **2.26×10^-4^** |
|  | Duration of fixation | **1.14×10^-11^** |  | 5.23×10^-1^ |  | 9.29×10^-2^ |
|  | **Number of saccades** | **6.75×10^-17^** |  | 1.00 |  | **5.44×10^-5^** |
|  | **Scanpath length** | **1.05×10^-24^** |  | 8.43×10^-2^ |  | **3.16×10^-3^** |
|  | Fixation density | **4.36×10^-15^** |  | 8.81×10^-2^ |  | 1.53×10^-1^ |
|  | **Number of blinks** | **7.19×10^-5^** |  | 1.00 |  | **3.14×10^-2^** |
| **Smooth pursuit test** | |  |  |  |  |  |
| ***Horizontal pursuit*** | |  |  |  |  |  |
|  | Horizontal position gain | **1.78×10^-5^** |  | 1.00 |  | 2.13×10^-1^ |
|  | Number of blinks | **3.20×10^-4^** |  | 1.00 |  | 5.59×10^-2^ |
| ***Slow Lissajous*** | |  |  |  |  |  |
|  | Horizontal position gain | **2.72×10^-7^** |  | 4.08×10^-1^ |  | 6.73×10^-1^ |
|  | Number of saccades | **1.59×10^-4^** |  | 1.00 |  | 1.47×10^-1^ |
|  | Peak saccade velocity | **2.45×10^-3^** |  | 7.16×10^-2^ |  | 1.00 |
|  | Horizontal velocity gain | **6.62×10^-5^** |  | 1.00 |  | 7.43×10^-1^ |
| ***Fast Lissajous*** | |  |  |  |  |  |
|  | **Horizontal position gain** | **7.99×10^-13^** |  | 1.00 |  | **1.51×10^-2^** |
|  | Vertical position gain | **1.29×10^-5^** |  | 1.00 |  | 2.43×10^-1^ |
|  | Number of fixations | **9.35×10^-7^** |  | 1.72×10^-1^ |  | 1.00 |
|  | Duration of fixations | **4.37×10^-5^** |  | 5.98×10^-2^ |  | 1.00 |
|  | Number of saccades | **7.88×10^-7^** |  | 3.44×10^-1^ |  | 9.02×10^-1^ |
| **Fixation test** | |  |  |  |  |  |
| ***No distractor*** | |  |  |  |  |  |
|  | Number of blinks | **5.33×10^-5^** |  | 1.00 |  | 3.83×10^-1^ |
| ***Near distractor*** | |  |  |  |  |  |
|  | Number of blinks | **8.52×10^-5^** |  | 1.00 |  | 2.18×10^-1^ |
| ***Far distractor*** | |  |  |  |  |  |
|  | Duration of fixation | **5.21×10^-4^** |  | 1.35×10^-1^ |  | 1.00 |
|  | Number of blinks | **2.67×10^-6^** |  | 9.71×10^-1^ |  | 4.29×10^-1^ |

The names of eye movement tests are written in bold and underlined script, paradigms within the tests are written in bold and italic script, and the obtained individual measures are written in smaller plain text. Bonferroni-adjusted *p* values are shown. The measures with significant differences between schizophrenia and autism spectrum disorder are shown in Bold script. HC: Healthy controls; SCZ: Subjects with schizophrenia; ASD: Subjects with autism spectrum disorder.
